# Open Release of Male Mosquitoes Infected with a *Wolbachia* Biopesticide: Field Performance and Infection Containment

**DOI:** 10.1371/journal.pntd.0001797

**Published:** 2012-11-15

**Authors:** Linda O'Connor, Catherine Plichart, Ayo Cheong Sang, Corey L. Brelsfoard, Hervé C. Bossin, Stephen L. Dobson

**Affiliations:** 1 Department of Entomology, University of Kentucky, Lexington, Kentucky, United States of America; 2 Institut Louis Malardé, Papeete, Tahiti, French Polynesia; The Pennsylvania State University, United States of America

## Abstract

**Background:**

Lymphatic filariasis (LF) is a globally significant disease, with 1.3 billion persons in 83 countries at risk. A coordinated effort of administering annual macrofilaricidal prophylactics to the entire at-risk population has succeeded in impacting and eliminating LF transmission in multiple regions. However, some areas in the South Pacific are predicted to persist as transmission sites, due in part to the biology of the mosquito vector, which has led to a call for additional tools to augment drug treatments. Autocidal strategies against mosquitoes are resurging in the effort against invasive mosquitoes and vector borne disease, with examples that include field trials of genetically modified mosquitoes and *Wolbachia* population replacement. However, critical questions must be addressed in anticipation of full field trials, including assessments of field competitiveness of transfected males and the risk of unintended population replacement.

**Methodology/Principal Findings:**

We report the outcome of field experiments testing a strategy that employs *Wolbachia* as a biopesticide. The strategy is based upon *Wolbachia*-induced conditional sterility, known as cytoplasmic incompatibility, and the repeated release of incompatible males to suppress a population. A criticism of the *Wolbachia* biopesticide approach is that unintended female release or horizontal *Wolbachia* transmission can result in population replacement instead of suppression. We present the outcome of laboratory and field experiments assessing the competitiveness of transfected males and their ability to transmit *Wolbachia* via horizontal transmission.

**Conclusions/Significance:**

The results demonstrate that *Wolbachia*-transfected *Aedes polynesiensis* males are competitive under field conditions during a thirty-week open release period, as indicated by mark, release, recapture and brood-hatch failure among females at the release site. Experiments demonstrate the males to be ‘dead end hosts’ for *Wolbachia* and that methods were adequate to prevent population replacement at the field site. The findings encourage the continued development and extension of a *Wolbachia* autocidal approach to additional medically important mosquito species.

## Author Summary

Additional tools are required to mitigate mosquito borne disease in the South Pacific, including human lymphatic filariasis (LF). *Wolbachia* are obligate intracellular bacteria that occur in a majority of insect species and that cause a form of conditional sterility in mosquitoes. Prior work demonstrates that male *Aedes polynesiensis* mosquitoes, which are artificially infected with *Wolbachia* (i.e., transinfected) can effectively sterilize wild type females in the laboratory, suggesting the potential applied use of *Wolbachia* as a pesticide for this medically important mosquito. As a critical intermediate step toward the development of the *Wolbachia* pesticide approach, we report on the field competitiveness of transinfected males and the risk of accidental horizontal transmission of *Wolbachia* from transinfected males. The outcome of laboratory cage trials and a thirty-week open release field trial provide evidence against horizontal transmission of *Wolbachia* from the transinfected males. Additionally, the field trial provides evidence for the competitiveness of transinfected males for indigenous female mates, as indicated by the failure of brood hatch and a resulting population level impact. No residual *Wolbachia* was detected in the targeted population during or after the male releases, showing released males to be ‘dead end hosts’ for *Wolbachia*. We discuss the results in relation to a disease control approach that integrates vector control with existing measures against LF.

## Introduction

Lymphatic filariasis (LF) is a disfiguring and socioeconomically burdensome disease estimated to affect over 120 million people worldwide, with 1.3 billion people at risk [1]. An ongoing global strategy for eliminating this mosquito borne disease is to interrupt transmission by administering annual macrofilaricidal prophylactics through mass drug administration (MDA) programs. However, in some regions the efficacy of these area-wide treatment programs can be compromised by the biology of the mosquito vectors.

In the South Pacific, the pattern of negative density dependent transmission displayed by the primary vector, *Aedes polynesiensis* makes this mosquito more efficient in low-level microfilaraemics [2], [3]. This complication has been hypothesized as a contributor to an inability to eliminate LF in the some areas of South Pacific, despite decades of ongoing MDA [2], [4]. As a result, augmentative vector control has been advised for areas where *A. polynesiensis* is the primary vector 1,4–7. Unfortunately, conventional vector control for *A. polynesiensis* has not been effective, due to the numerous, cryptic and inaccessible breeding sites of this mosquito and the geography of the Pacific Islands, which hinder control efforts due to the difficult logistics of moving control personnel and equipment between islands, even in those countries with relatively well-developed vector control programs [6], [8].

Prior laboratory and field cage trials have examined an autocidal approach based upon artificial infections of *Wolbachia* [9], [10], an obligate intracellular bacterium estimated to occur in a majority of insect species [11]. In mosquitoes, *Wolbachia* causes cytoplasmic incompatibility (CI), which can lead to arrested embryonic development in populations that include individuals infected with different *Wolbachia* types. Bidirectional CI results in egg hatch failure in both cross directions and was the basis of a prior, successful suppression of a Culex quinquefasciatus population in Burma [12]. In brief, the approach is similar to the Sterile Insect Technique (SIT) [13]–[15] in which repeated, inundative releases of sterile males act to sterilize females in the targeted field population. Releasing male mosquitoes does not pose a health threat, since they do not blood feed or vector disease. The released males are also ‘dead end hosts’ for the maternally inherited *Wolbachia*, so that the released infection type does not become established in the field. Despite the successful prior field trial, the *Wolbachia*-based suppression approach was considered an isolated demonstration, since naturally occurring bidirectionally-incompatible populations are rare [16]. Recently however, the development of methods for the artificial generation of bidirectionally-incompatible mosquito strains permits broader application [10], [17], [18]. Natural populations of *A. polynesiensis* are infected with a single *Wolbachia* type [19]–[21]. In 2008, an artificially infected *A. polynesiensis* strain (CP) was generated by introgressing an alternate *Wolbachia* type originating from *A. riversi* into the *A. polynesiensis* genotype. The resulting CP males of the *Wolbachia* transfected strain of *A. polynesiensis* are incompatible with wild type females and show mating competitiveness equal to that of wild type males in laboratory trials [9], [10].

The fitness/competitiveness of released males is a critical component of SIT approaches, including both traditional irradiation-based sterility [22] and newer transgenic approaches [23], [24]. Prior experiments within cages demonstrate good fitness of the CP males relative to the wild type males, with a high competitive index (C) (C>0.8) [9]. But prior to full-scale field trials (e.g., intended to suppress and eliminate populations), competitiveness must be assessed in the field.

An additional objective of the open release trial was to assess the risk of unintended population replacement [18], [25]–[27]. While population replacement is a desired outcome in some *Wolbachia*-based strategies [28] and a potential goal for downstream strategies with CP [10], it was not the goal here. In the *Wolbachia*-based suppression strategy, the establishment of the artificial *Wolbachia* type in the targeted population could allow compatibility and reduce the suppressive effect of CP male releases. Horizontal movement of *Wolbachia* at an evolutionary time scale is hypothesized, based upon prior phylogenetic studies [29]. However, it is unclear what role male hosts play in horizontal movement.

## Materials and Methods

Mosquitoes were reared in the laboratory at the University of Kentucky using previously described methods [30]. To examine for paternal transmission of *Wolbachia* to incompatible *A. polynesiensis, A. albopictus, or A. aegypti* 150–200 virgin females of each species were released into a 1×1×1 m cage containing 350–400 CP males. Control crosses for female fertility consisted of 10 males and 10 females of CP, *A. polynesiensis*, *A. albopictus*, or *A. aegypti*. Adults were provided a 10% sucrose solution. Female mosquitoes were blood fed using mice for 20 min. Weekly, females were provided oviposition substrate. Eggs were allowed to mature for 7–10 d on a damp oviposition substrate. Eggs were hatched in 700 ml of a 1∶1 solution of 6 g/ml liver powder and deionized water. Control cages were closed following one gonotrophic cycle, after showing females were fertile by examining for hatching eggs. *Wolbachia* A-type and B-type infections were tested for using the wsp primers, 136/691R and 81F/522R, respectively [31]. DNA was extracted by emulsifying whole adult mosquitoes in a 1.5 ml Eppendorf tube containing 100 ul of buffer containing 10 mM Tris-HCl, 1 mM ethylendiaminetetraacetic acid (EDTA), and 50 mM NaCl, at pH 8.2, using a Mini-bead beater (Biospec Products, Bartlesville, OK). After homogenization, samples were incubated at 100°C for 5 min and centrifuges at 16,000× g for 5 min. PCR was conducted as described previously [31].

CP males were reared using previously described, laboratory methods [10], [30] at the Institute Louis Malardé on Tahiti, French Polynesia. Individuals for release were separated by sex using a previously defined mechanical method that separates by sexual size dimorphism [32] using a device manufactured by the John W. Hock company (Gainsville, FL). On average, the mechanical sorting method removed approximately 90% of females. Following eclosion, the mechanically sorted male pool was visually examined to remove the remaining females. Males were transported in a cooler (Model no. 5205A773, Coleman, USA) pre-chilled to 15°C via commercial flight to Raiatea and carried by boat to TOA for release. A U12 Hobo data logger (OnSet, USA) was used to monitor temperature and humidity during the mosquito transports. Average temperature and humidity were 14.5±0.7°C and 55±6% RH (mean±s.d.), respectively. In total, 6 hours were required for transport.

Monitoring of the adult population occurred at a two-week interval via BG traps (Biogents, Regensburg, Germany) before, during, and after CP male releases. Three BG traps were placed at separate locations that were evenly distributed across each island, with collections being made for a 20-minute period.

To measure egg hatch, gravid females were individualized in oviposition containers. The resulting eggs were submerged to hatch and then observed for any resulting larvae. Spermatheca were dissected from females, crushed in a solution of PBS on a microscope slide using a coverslip and then examined using a compound microscope [33].

PCR detection of *Wolbachia* was based upon previously described protocols [10], [19], [20], [30] using the 136F/691R wsp primers to detect A-type and 81F/522R wsp primers to detect B-type *Wolbachia*. DNA extraction was performed on pooled mosquitoes with heads removed, using the Qiagen DNeasy kit (Qiagen, Valencia, CA). Elution was in 200 µl and 5 µL DNA was used for PCR. The PCR assay was performed by using the iQ SYBR Green Supermix and an iCycler iQ Thermocycler (Bio-Rad, Hercules, CA).

A Before-After-Control-Impact-Paired-Series (BACIPS) statistical design was used to examine for an impact of CP male releases. The BACIPS approach is designed to compensate for differences between the release and no-release sites, as well as temporal variance [34]–[36]. T-test comparisons with Bonferonni correction were performed for delta values for the fourteen collections immediately prior to CP male releases (‘Before’) and the fourteen collections occurring during releases (‘During’), with delta values determined using the following formula:
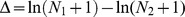
where N_1_ and N_2_ are the numbers of adult females collected at Sites 1 and 2, respectively. Comparisons were of all combinations of the two no-release sites (HOR, ANO) and the release site (TOA) receiving CP males.

All statistics were performed using JMP 8.0.1 (SAS Institute Inc.).

### Ethics Statement

The importation of the CP strain and subsequent release of CP males were permitted via French Polynesia Ministry Council decision n° 1392 CM, Oct 17, 2007. Field-work conducted on private land was with permission from the owners. The use of laboratory mice (*Mus musculus*) at the Institut Louis Malardé was approved by the “Commission permanente de l'assemble de la Polynesie Francaise (Tahiti)” [Deliberation#2001-16/APF]. Animal work at the University of Kentucky was approved by the Institutional Animal Care and Use Committee 00905A2005).

## Results

To assess the risk of horizontal transmission of *Wolbachia* from CP males, large laboratory cage assays were performed prior to open field releases. CP males were added to cages containing virgin *A. polynesiensis*, *A. albopictus* and *A. aegypti* females. As shown in Table 1, control crosses of intraspecific matings demonstrated good fertility of females (>50% egg hatch). While females continued to produce eggs in the interspecific matings, low egg hatch was observed, with only three of >25,000 eggs hatching. Of the three resulting larvae, two survived to adult, and both were *A. polynesiensis* males. PCR assays showed both males to be infected with the wild type *Wolbachia*. Thus, the F1 individuals were from rare egg hatch that results from *A. polynesiensis* females that are incompatibly mated with CP males [9], [19].

**Table 1:** Egg hatch resulting from intra- and inter-specific crosses.

	Egg Number		Percent
	Unhatch	Hatch	Hatch
**Interspecific Crosses** *			
**Replicate 1**	10,115	1	0.010%
**Replicate 2**	15,496	2	0.013%
**Intraspecific Crosses**			
***A. polynesiensis, CP Strain***	190	273	59.0%
***A. polynesiensis, Wild Type***	5	91	94.8%
***A. aegypti***	2	598	99.7%
***A. albopictus***	57	163	74.1%

* Interspecific crosses consist of CP males combined with virgin female.

*A. polynesiensis*, *A. aegypti* and *A. albopictus*.

For field releases of CP males, the sites were ‘motu’ islands, selected due to their small size, isolation and absence of human inhabitants. Prior characterization of the *A. polynesiensis* populations demonstrate the targeted motu to be infested with unusually large populations, more than one hundred times more dense than sites on the adjacent mainland [37]. This large population size makes the motus unattractive locations for early population suppression attempts. However, their isolation and prior characterization make them useful for examining questions of male competitiveness and replacement risk.

**Figure 1:**
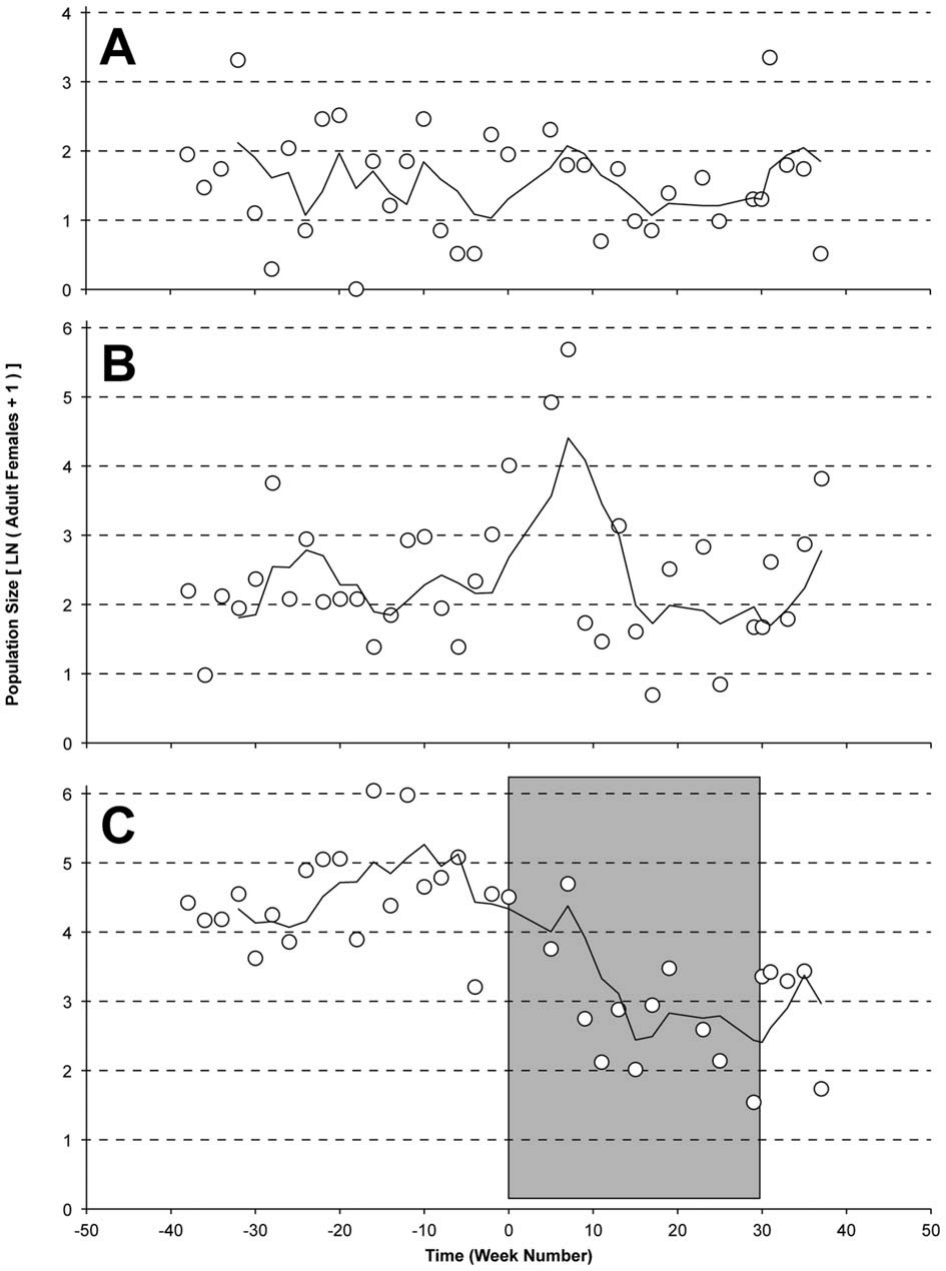
*A. polynesiensis* population dynamics. Collection data is shown for the A) Tiano (ANO), B) Horea (HOR) and C) Toamaro (TOA) study sites, as measured by BG trap collections of adult females. Lines show moving averages across four collection periods. Time is shown as the relative week number, with ‘Week 0’ as the start of releases. The grey shaded box indicates the release period on TOA, with CP releases ending on Week 30.

Prior to the start of CP releases, a standardized collection protocol was used to monitor adults from the sites intended as release and no-release locations (Fig. 1). Monitoring at the three sites was ongoing for more than a year prior to the release start [37]. The highest population densities of *A. polynesiensis* were observed on TOA (166±209, n = 96; Avg ± StDev adult females, number of collections) and HOR (96±157, n = 76). A lower population density was observed on ANO (12±14, n = 76), which received substantial source reduction activity by the landowner. The population densities were seasonally variable, and capable of reaching high densities, with a maximum of 1,260 *A. polynesiensis* females collected in a 20-minute period at TOA in late August of 2009.

Beginning in December 10, 2009, the TOA site received an average of 3,800 CP males/week. CP males were reared on Tahiti and transported to Raiatea for release. CP male releases continued for thirty weeks, with more than 117,000 CP males released in total. There is no marker that is transferred from *Wolbachia* in the male to the mate that can be detected in mated females. Therefore, we relied upon an indirect measure to assess CP male competitiveness in the field: the likelihood of a female producing a non-hatching brood. Females collected at the TOA and HOR sites were isolated and allowed to oviposit, and egg hatch was recorded. During the period in which CP males were released, the proportion of a female producing hatching eggs was significantly lower at TOA relative to HOR, X^2^ (1, N = 887) = 38.18, p<0.0001. In contrast, females at the release and no-release sites were equally likely to produce hatching eggs both before the start of CP male releases, X^2^ (1, N = 141) = 2.22, p = 0.13 and following the termination of releases, X^2^ (1, N = 154) = 0.49, p = 0.48 (Table 2). An analysis of the same data, comparing the different trial phases (‘no release’ versus the ‘during release’ periods) within a site shows no difference for HOR, X^2^ (2, N = 412) = 4.69, p = 0.096 and a significant difference at TOA, X^2^ (2, N = 770) = 44.33, p<0.0001.

**Table 2:** Percent females that produced hatching egg broods at a no-release site (HOR) and the site receiving CP male releases (TOA).

	Field Trial Phase		
	Before	During	After
**TOA**	100%	76%*	97%
**HOR**	98%	93%	99%

The asterisk indicates a significant difference X^2^ (1, N = 887) = 38.18, p<0.0001.

The failure of females to produce hatching eggs at the release site could result from cytoplasmic incompatibility or a lack of insemination. To examine for the latter, field collected females were dissected to examine spermatheca. High rates of fertilization were observed throughout the study at both the release site (88% fertilized; n = 350 females) and no-release site (85% fertilized; n = 231) sites, and no difference was observed between the sites, X^2^ (1, N = 581) = 0.72, p = 0.39.

Male competitiveness can be estimated based upon the number of released CP males, the estimated number of wild type males and the frequency of incompatible mating events. Existing collecting methods yield low numbers of *A. polynesiensis* males on Toamaro [37], [38]. Therefore, a mark release recapture experiment was performed at the start of CP male releases. CP males were marked with DayGlo, released and recaptured as previously described [38]. Collection using backpack aspiration yielded a total of 96 males in the three days of sampling, five of which were recaptured males. A modified Lincoln index was used to estimate male population size [39], [40],
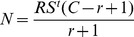
where N = estimated population density on day t , S = estimated probability of daily survival [41], R = number of released females, C = number of captured females, r = number of recaptured females.

Across the three recapture days, the male population size was estimated at approximately 5,900 males. Thus the 2,162 marked and released CP males represented approximately 37% of the indigenous male population size.

Using a previously defined index [42], the field competitiveness (C) was estimated from the estimated number of indigenous males (N) and incompatible males (S),
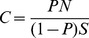



The proportion of incompatible matings (P) was estimated at 0.2, based upon measurements of female incompatibility on Toamaro (Table 2). Using this definition, the competitiveness of CP males is estimated at 0.68, where 1.0 would be equivalent fitness with wild type males. Relative to analogous estimations of classical, irradiation based SIT and newer transgenic approaches, this represents a relatively good level of competitiveness [24].

**Figure 2:**
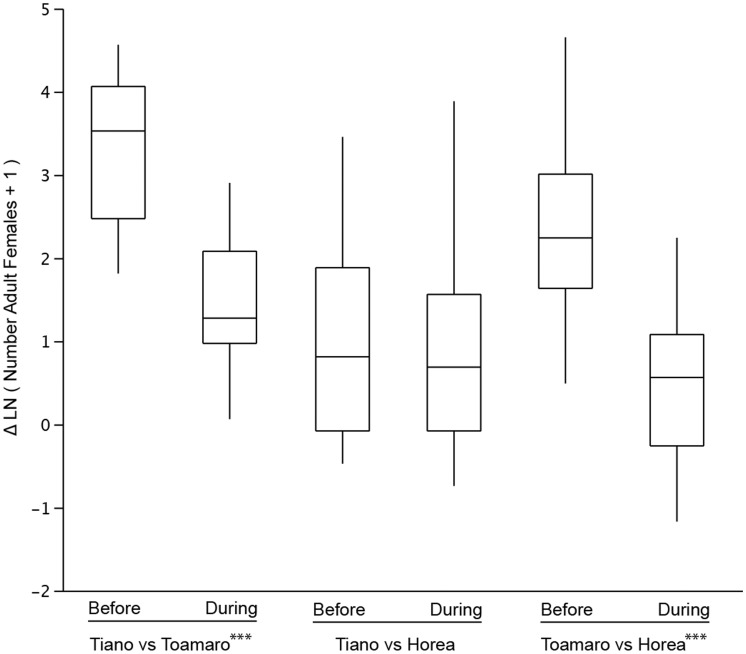
Box plots of delta values used in the BACIPS statistical analysis. Each of the three possible combinations of site pairs is shown. For each pair, delta values are of collections within the thirty-week period immediately prior to the start of CP male release (‘Before’) and the thirty-week period during CP male release (‘During’). Delta values are calculated as the difference between population numbers at the sites, with population number indicated as ln(*Female Number* +1). Sites are the release site Toamaro (TOA), which received releases of CP males, and the two no-release sites Tiano (ANO) and Horea (HOR), which did not receive CP male releases. Asterisks indicate a significant difference in comparisons of the ‘Before’ and ‘During’ release periods (p<0.0001).

Due to the low proportion of incompatible males on Toamaro, it was not clear that population-level impacts would result from the CP male releases. To examine for an effect of CP male releases on the targeted *A. polynesiensis* population, a statistical method developed for environmental impact assessment was used, known as Before-After-Control-Impact-Paired-Series (BACIPS) [34]–[36]. Pair-wise comparisons were performed for the population size (i.e., number of adult females) for the ‘before release’ and the CP male ‘during release’ periods, including all combinations of the two no-release sites (HOR and ANO) and the release site (TOA). Comparison of the two no-release sites indicated no difference between the two time periods, t(25) = 0.03, p = 0.51. In contrast, comparisons of the release site (TOA) showed a significant difference between the ‘before’ and ‘during’ periods for pairwise comparisons with both HOR, t(25) = −4.72, p<0.0001 and ANO, t(25) = −5.67, p<0.0001 (Fig. 2).

## Discussion

Horizontal transfer of infection from males did not occur in laboratory experiments. These results provide evidence against the ability of CP males to transmit *Wolbachia* to conspecific and congeneric females under conditions of close proximity and probable interaction and are consistent with prior experiments examining for horizontal transfer to predators [43]. A sustained open release of CP males provides an additional test for horizontal transfer. Furthermore, an additional route for unintended population replacement is via the accidental release of CP females. To examine for establishment of the CP *Wolbachia* type in the field (i.e., either by accidental CP female release or paternal transmission), females were collected from TOA (n = 83 females) and HOR (n = 30 females) populations throughout the study, ending in August 2010, following the termination of releases. The presence of the wild type *Wolbachia* and absence of the CP male type *Wolbachia* was observed in all field-collected females [10].

The results demonstrate that laboratory reared, sorted, and delivered CP males survive and competitively mate with indigenous *A. polynesiensis* females within a field population. Despite the relatively small numbers of released males relative to the large indigenous population size, we observed a significant decrease in the number of TOA females able to produce viable embryos. In contrast, decreases were not observed at the two control sites, where CP males were not released. This observation supports that the observed decrease in egg hatch was due to CP male releases and not seasonal and weather driven events.

In addition to the laboratory tests, the results of the open CP male releases showing the absence of the B-clade *Wolbachia* are also consistent with the hypothesized role of males as ‘dead end hosts’ for *Wolbachia*. Specifically, we have observed no evidence for the introduced *Wolbachia* type persisting on TOA outside of the released CP males, despite maintaining a sustained presence of CP males on TOA for more than 200 days and releasing more than 100,000 CP males. We note that, even with the introduction of a CP female into a population, the outcome may not be the establishment of the B-type *Wolbachia*. If a CP female were released, she must mate with a compatible male, blood feed and successfully oviposit. For the infection to become established, any resulting progeny must survive and compete successfully against wild type conspecifics. As described above, sons are unlikely to transmit *Wolbachia*. Daughters are expected to inherit the B-type *Wolbachia*, but must mate with compatible males and survive to oviposit. Prior comparisons show that CP immature and adult females display lower fitness relative to wild type mosquitoes [30]. The results support the continued development of additional methods in support of larger downstream applications. In particular, improved sex-separation tools can simplify the production process and reduce overall costs. This can include the development of methods to ‘inactivate’ any females that are unintentionally released [22].

The results show that following mass production, sex separation and delivery, CP males are competitive mates under field conditions. Existing methods were adequate for biological containment of the released *Wolbachia* type. An impact on the targeted population was observed despite relatively small release numbers. The results are consistent with traits desired for an IIT approach and encourage additional trials in which CP males are released at a larger scale and at an epidemiologically relevant site. Furthermore, the results support the continued development and expansion of the IIT approach to additional medically important systems [17].
